# Synergistic impacts of habitat loss and fragmentation on model ecosystems

**DOI:** 10.1098/rspb.2016.1027

**Published:** 2016-09-28

**Authors:** Lewis J. Bartlett, Tim Newbold, Drew W. Purves, Derek P. Tittensor, Michael B. J. Harfoot

**Affiliations:** 1 Centre for Ecology and Conservation, College of Life and Environmental Sciences, University of Exeter, Penryn Campus, Penryn, UK; 2 United Nations Environment Programme World Conservation Monitoring Centre, Cambridge, UK; 3 Computational Science Laboratory, Microsoft Research, Cambridge, UK; 4 Dalhousie University, Halifax, Nova Scotia, Canada

**Keywords:** habitat fragmentation, habitat loss, land-use change, trophic shift, biomass pyramid, ecosystem response

## Abstract

Habitat loss and fragmentation are major threats to biodiversity, yet separating their effects is challenging. We use a multi-trophic, trait-based, and spatially explicit general ecosystem model to examine the independent and synergistic effects of these processes on ecosystem structure. We manipulated habitat by removing plant biomass in varying spatial extents, intensities, and configurations. We found that emergent synergistic interactions of loss and fragmentation are major determinants of ecosystem response, including population declines and trophic pyramid shifts. Furthermore, trait-mediated interactions, such as a disproportionate sensitivity of large-sized organisms to fragmentation, produce significant effects in shaping responses. We also show that top-down regulation mitigates the effects of land use on plant biomass loss, suggesting that models lacking these interactions—including most carbon stock models—may not adequately capture land-use change impacts. Our results have important implications for understanding ecosystem responses to environmental change, and assessing the impacts of habitat fragmentation.

## 1. Introduction

Land-use change is a major driver of terrestrial biodiversity loss [1–3], and is predicted to increase in importance as global development continues [4,5]. Multiple aspects of land-use change have been identified as drivers of population collapses and extinction [6]. These aspects include habitat loss (outright removal of habitat patches), habitat degradation (reduced quality of habitat patches), and fragmentation (reduced functional connectivity of patches across a landscape). Many studies have concluded that habitat loss is a greater threat to biodiversity than fragmentation—reviewed in Villard & Metzger [7]. However, extinction may be underestimated when fragmentation is ignored, for example using species–area relationships [8–10]. Habitat fragmentation usually accompanies habitat loss, and disentangling their effects remains challenging [11,12]. Consequently, the use of the term ‘habitat fragmentation’ to encapsulate both habitat loss and habitat configuration has been questioned [13]. The usefulness of the term ‘habitat fragmentation’ arguably relies on a strong interdependence of the effects of habitat loss and fragmentation [14]. This interdependence is a question of how different aspects of land-use change interact.

The host of interacting factors involved makes predicting specific effects of land-use change challenging [15]. Ecology traditionally favoured an approach where model simplicity is valued [16]. Whole-ecosystem models, at least in the terrestrial realm, accounting for trophic interactions, have typically lacked spatially explicit dynamics [17], and population models accounting for spatial structure have been criticized for focusing on single species [18]. There is a need to combine these approaches, with mounting evidence showing the importance of interacting factors in determining ecosystem responses, including demography, spatial structure, and climate [19–21]. Furthermore, changes in land use do not affect all species equally; sensitivity to both habitat loss and fragmentation varies with species' numerous ecological traits [22–30].

To study these potential interactions, and to complement simpler approaches, more complex models with greater ecological realism are helpful, so long as increasing complexity adds predictive value [31]. Our study aims to examine the importance of the possibly complex interactions between habitat loss and habitat fragmentation. We use a general ecosystem model—the Madingley model—to simulate ecosystem responses to multiple land-use scenarios. The Madingley model can reproduce the structure of ecological communities at broad spatial scales, with ecosystems being emergent and dynamic [32]. We capitalize on the Madingley model's trait-based and spatially explicit simulations to investigate how habitat loss/degradation and fragmentation might interact. We look for evidence of synergies between aspects of land-use change. Additionally, we look for how trait-based susceptibility to land-use change may exacerbate ecosystem impacts. For example, heightened vulnerability of specific feeding types may distort coarse biomass ratios in trophic pyramids. Finally, we also examine potential top-down effects of higher trophic levels on the response of plant biomass to land-use impacts.

## 2. Material and methods

### (a) The model system

The Madingley model is a general ecosystem model that attempts to include the complete autotroph and heterotroph structure of ecosystems with dynamically assembling communities. It is flexible in spatial extent and resolution, with abiotic environmental variables based on simulated real-world locations. We used only the terrestrial capabilities of the model. We provide a summary below, but refer to Harfoot *et al.* [32] for a full model description.

Organisms are defined by functional traits, rather than taxonomically. Heterotrophs are modelled as individuals, defined by both categorical and quantitative traits. Categorical traits are: trophic group (carnivore, omnivore, herbivore); thermoregulation strategy (endotherm, ectotherm); and reproductive strategy (semelparous, iteroparous). Quantitative traits are: current body mass; mass at birth; and mass at reproductive maturity. Current body mass contributes to calculating metabolic rates, mortality, dispersal distances, feeding rates, and optimal prey size, based on allometric relationships encoded in the model. Body mass does not directly determine the spatial ecology of heterotrophs other than dispersal distances—habitat requirements are an emergent outcome of trophic interactions, metabolic expenditure, and modelled landscape characteristics. Because it is computationally unfeasible to model each individual organism separately [33], individuals are grouped into ‘cohorts’—collections of organisms with the same functional traits—that are treated identically in the model. Autotrophs are modelled after Smith *et al.* [34], and are represented as biomass pools. Autotrophs can be deciduous or evergreen, and vary their relative investments in structural and leaf biomass, with only leaf biomass available for herbivory.

The dynamics of individuals, represented by heterotroph cohorts and autotroph pools, is modelled in one-month time steps. Within each time step, the model simulates the autotroph ecological processes of growth (photosynthesis based on local net primary productivity) and mortality (herbivory and climate driven); and heterotroph metabolism, eating (herbivory and predation), reproduction, growth, mortality, and dispersal. Dispersal has two components—natal dispersal after birth and responsive dispersal triggered by starvation. Dispersal distances are determined by body mass, with larger animals dispersing further. See electronic supplementary material, figure S1 for a representation of ecological processes. The model reaches a dynamic steady state within 100 years (1 200 time steps) [32].

**Figure 1.**
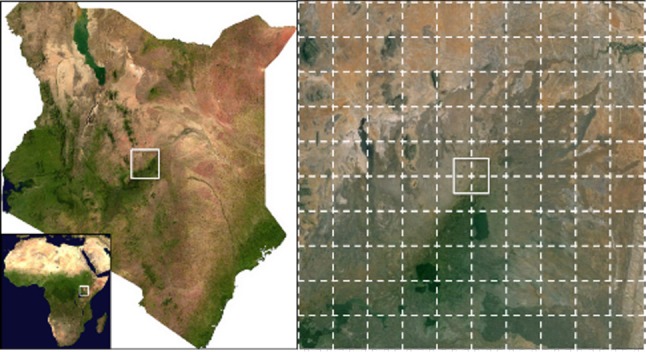
Maps show the location and scale of simulations. Right-side figure is the 1° × 1° large-scale landscape, with the small-scale 0.1° × 0.1° landscape at the centre of the image. Dashed lines represent the 100 cells of the large-scale simulations, the solid square represents the extent of small-scale simulations, which also contained 100 cells.

We simulated a habitat landscape at two scales: a 10 × 10 grid of either 0.1° or 0.01° grid cells in North Meru, Kenya (grid centre 0.05° N, 38.00°E; figure 1). We selected this location as an area where large megafauna are still extant and numerous, better reflecting our simulated ecosystems in which large-bodied (more than 100 kg) heterotrophs persist. The two scales, approximately 123 and 12 300 km^2^, fall within the size range of Kenyan national parks which support large-bodied species, e.g. Nairobi National Park at 117 km^2^, Tsavo East/South Kitui National Park contiguous at 13 580 km^2^ [35].

The habitat within each cell is approximated as homogeneous. Cells are connected to each other by dispersal, with differences in cell habitat and distances between cells approximating the real-world habitat landscape. The simulated landscape was bounded, with no migration into or out of the 10 × 10 grid. All simulations were repeated at the two scales, which we term ‘large scale’ and ‘small scale’. The natural habitat type of each cell emerges based on the abiotic environment (temperature and soil moisture [34]), e.g. coarse differences between dominance of trees or grasses are captured by different rates of investment in structural or leaf biomass.

### (b) Land-use scenarios

The scenarios considered represented combinations of different intensities, extents, and spatial configurations of human impact on vegetation. Lower intensities are analogous to habitat degradation, whereas the higher intensities represent habitat loss. The extent of land-use change represents how much habitat is degraded or lost. The spatial configuration of impacts was ‘random’ or ‘continuous’, capturing whether habitat is fragmented in the simulations (random) or contiguous (continuous). In random simulations, we selected cells randomly for disturbance, whereas in continuous simulations, we selected cells in rows maintaining unbroken areas of impacted and pristine habitat. While habitat fragmentation in the real world is rarely random, the resolution of the simulations (number of grid cells) required to accurately recreate specific fragmentation patterns was computationally intractable. To simulate different intensities of land-use change, we removed fixed proportions (25%, 50%, 75%, 100%) of the autotroph biomass in impacted cells at each time step. To simulate different extents of land-use change, we removed autotroph biomass from different proportions (25%, 50%, 75%, 100%) of cells. These treatments are summarized in electronic supplementary material, table S1 and an example diagram is shown in electronic supplementary material, figure S2. Each simulation was repeated 10 times, resulting in 580 simulations (10 replicates × 29 treatments × 2 spatial scales).

Each simulation was run without impact for 100 years to reach a ‘steady-state’ ecosystem [32]. Simulations were initialized with heterotroph populations drawn from the full range of possible functional characteristics detailed in Harfoot *et al.* [32], with a subset of these persisting to the steady-state ecosystem described. The range of body sizes for heterotrophs seeded into the simulations spanned 0.4 mg to 5 000 kg, with endothermic herbivores of body mass approximately 900 kg being the largest to persist to steady-state ecosystems at both large and small scales. From this baseline, the simulations were then run for a further 100 years under the appropriate land-use scenario. We performed all of our statistical analysis on the final 10 years of the simulation.

Details of the parameters and initialization files required to recreate these simulations can be found in electronic supplementary material, S2. All fundamental ecological parameters used in the model were unchanged from the version of the Madingley model published in Harfoot *et al.* [32], which have been tested to recreate empirical ecological processes (electronic supplementary material, S2 and table S5). The different scenarios on which this study is based are detailed by two initialization files required to run the Madingley model. Different spatial configurations, impact extents, and impact intensities are detailed in the ‘Scenarios.csv’ file, of which we provide a transcript (electronic supplementary material, S2 and table S3). The two simulated scales are detailed in the ‘EcosystemModelInitialisation.csv’ file, of which again we provide an annotated transcript (electronic supplementary material, S2 and table S4). The majority of values in electronic supplementary material, table S4 were unmodified from the previously tested and published version of the Madingley model, or if changed pertain to practical details of running the simulations. We highlight in electronic supplementary material, table S4 which aspects are relevant to the design of this study.

### (c) Information extracted for analysis

As a summary measure of ecosystem change, we calculated trophic skew. This metric, which we devised, compares the relative proportions of carnivore, omnivore, herbivore, and autotroph biomass between pristine reference ecosystems and those experiencing land-use change. Our pristine reference ecosystems are the emergent ecosystems we see in simulations where no plant biomass was extracted (electronic supplementary material, table S1). We calculated it using the following equation.2.1
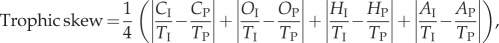
where *C*, *O*, *H*, *A*, and *T*, represent carnivore, omnivore, herbivore, autotroph, and total biomass, and *I* and *P* represent ‘impacted’ or ‘pristine’ scenarios.

We used this metric for two reasons. First, this metric reflects substantial ecological shifts—for example, widespread replacement of carnivores by omnivores, or release of predation resulting in greater herbivore and reduced autotroph biomass. Second, the metric captures strong biases against trophic groups; the loss of an equal proportion of biomass from each trophic group would yield a trophic skew of 0. Therefore, it indicates whether there are trait-dependent responses to our scenarios. We excluded complete ecosystem collapse from this metric (extent and intensity both 100%).

To test the importance of trophic-mediated ecosystem responses, we focused on autotroph biomass. We made a naive prediction of how much autotroph biomass would remain in the system after we remove a proportion of it through the impact scenario. For example, in a scenario of 50% extent and 50% intensity (approx. 25% biomass removal), if higher trophic levels were unimportant, then we would naively predict that the impacted system would have 75% of the autotroph biomass of a pristine system. We calculated the proportional difference between the naively predicted biomass and the biomass observed in the simulations using equation (2.2). This value could be positive (indicating a release from herbivory) or negative (indicating increased herbivore pressure, or failure to regenerate lost biomass before further removal). The magnitude of the value indicates the relative importance of potential trophic effects relative to the severity of land-use change.2.2
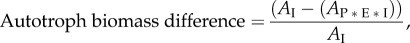
where *A* represents autotroph biomass, *I* and *P* represent ‘impacted’ or ‘pristine’ scenarios, and *E* and *I* represent ‘extent’ and ‘intensity’ (or, the proportion of cells impacted, and the proportion of autotroph biomass removed from impacted cells, respectively).

To understand which heterotroph traits were important determinants of response, we looked at population prevalence across a subset of our scenarios. Our trophic skew metric-captured coarse differences in response to habitat loss, degradation, and fragmentation. For more detailed analyses, we investigated the effect of complete loss of habitat patches (100% impact intensity) on populations in remaining ‘pristine’ patches. This allowed us to label habitat patches as ‘suitable’ or ‘unsuitable’, without further assumptions about patch quality. We characterized heterotroph response as the number of patches containing populations compared with the total number of remaining pristine patches.

### (d) Statistical approach

Our analyses employed a frequentist approach to understand ecosystem responses to our different scenarios, in the same way an empirical study might. Simulation studies using this approach may find an overabundance of significant differences [36], and so the most informative parts of our analyses were the examination of relative effect sizes, coefficients, *R*^2^-values, and means of differences (m.o.d.). Our results are best understood in terms of these values. We present *p*-values as part of the comprehensive reporting of our statistical analyses, in line with published guidance [37], but stress that they should not be considered in isolation.

We conducted initial analyses using generalized linear models (GLMs). We successfully normalized our metric data (trophic skew, autotroph biomass difference) through an arcsine transformation [38] and used a Gaussian error structure, undertaking backward stepwise model selection to find minimum adequate models [38]. We tested for complex three- and four-way interactions as we had good *a priori* reasons to consider them, for example: (i) the effect of increasing impact intensity could depend on the spatial extent of impact, (ii) this interaction could depend on spatial configuration, and (iii) these interactions may be different at our two simulated scales. Our minimum adequate models remained very complex (electronic supplementary material, S1); to interpret the interactions, we analysed subsets of the data—controlling for specific dimensions of variation, using linear regressions and *t*-tests. We performed four linear regressions of intensity for our four different extents, and vice versa. We compared our two scales and two configurations using paired *t*-tests, to match otherwise equivalent scenarios. We did not correct for multiple testing, as these subset tests were used to interpret established differences in simulations, and as previously discussed, the values most informative for interpretation relate to effect sizes.

To analyse heterotroph responses (number of pristine cells remaining inhabited by particular functional groups), we focused on a subset of the simulations, only examining those with 100% impact intensity, and considered our two scales separately. We employed backwards stepwise model selection on GLMs [38], using a quasi-binomial error structure to correct for overdispersion in our presence/absence data [39]. The minimum adequate models again showed complex three-way interactions (electronic supplementary material, S1). We further subset the data by spatial configuration, and used generalized linear mixed-effects models to better interpret our results. We set trophic group as a random effect to generalize mass response across trophic groups. We used a binomial error structure to allow us to use mixed-effects models, as again this subset analysis should be understood in terms of the coefficients presented rather than *p*-values.

## 3. Results

### (a) Trophic skew

**Figure 2.**
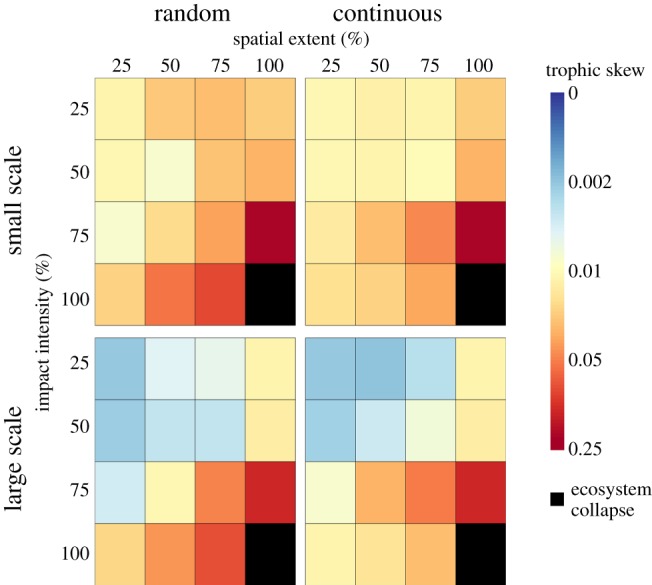
Mean trophic skew across different impact scenarios, logarithmically colour coded. Panels are split by simulation scale and spatial configuration. Black cells represent total ecosystem collapse.

Mean trophic skew across all simulations (figure 2) equalled 0.148 but varied across scenarios by approximately two orders of magnitude. Increasing intensities and extents of autotroph removal resulted in greater degrees of trophic skew for both scales (figure 2). There were complex interactions governing this effect (electronic supplementary material, S1). We undertook linear regressions on subsets of the data to illustrate more clearly these interactions (table 1). Across both scales, greater trophic skew was seen with increasing spatial extent at any given impact intensity, and with increasing impact intensity for any given spatial extent (figure 2 and table 1). The explanatory power of increasing extent and intensity (*R*^2^-values, table 1) was generally high. Effects of the intensity of impact were generally stronger when the extent of impact was greater, and vice versa, indicating that the effects of extent and intensity are synergistic (*β*-values, table 1).

**Table 1.** Regression analyses performed on data subsets. Subset value represents the value of either extent or intensity for that subset, depending on which variable the regression was performed on. *β* is the regression coefficient for trophic skew as a function of either extent or intensity (see column headings).

	large scale								small scale							
	extent regression (subset by intensity)				intensity regression (subset by extent)				extent regression (subset by intensity)				intensity regression (subset by extent)			
subset value (%)	*β*	*R*^2^	d.f.	*p*	*β*	*R*^2^	d.f.	*p*	*β*	*R*^2^	d.f.	*p*	*β*	*R*^2^	d.f.	*p*
25	0.072	0.371	78	<0.001	0.11	0.726	78	<0.001	0.054	0.197	78	<0.001	0.042	0.31	78	<0.001
50	0.085	0.599	78	<0.001	0.17	0.654	78	<0.001	0.1	0.442	78	<0.001	0.099	0.31	78	<0.001
75	0.426	0.923	78	<0.001	0.276	0.659	78	<0.001	0.493	0.74	78	<0.001	0.179	0.538	78	<0.001
100	0.223	0.434	78	<0.001	0.56	0.741	78	<0.001	0.235	0.494	78	<0.001	0.692	0.67	78	<0.001

Higher values of trophic skew were generally seen in the random spatial configuration (figure 2). Our GLMs again highlighted an interaction effect (electronic supplementary material, S1). The greater effect of random spatial configuration versus continuous was present at both scales, but stronger at the small scale (paired *t*-tests; *large: t*_119_ = 2.60, *p* = 0.010, m.o.d. = 0.0134; *small: t*_119_ = 4.81, *p* < 0.001, m.o.d. = 0.0244). The magnitude of this difference can be seen by examining mean pairwise differences, with random configurations yielding trophic skews 18.1% higher than continuous equivalents (*large:* 16.0%, *small:* 20.2%), in line with the m.o.d. values relative to the mean trophic skew. Overall, small-scale simulations showed significantly higher values of trophic skew across scenarios compared with large-scale simulations (paired *t*-test, *t*_299_ = 15.95, *p* < 0.001, m.o.d. = 0.0431). Expressed as a percentage difference between equivalents, small-scale simulations yielded skew values 69.4% higher than large-scale equivalents. The large size of this effect when expressed as a percentage appears at odds with the m.o.d., and is a consequence of the nonlinear response of trophic skew to increasing extent and intensity (figure 2). Comparing between scales, less severe scenarios differ by an order of magnitude in trophic skew values, but these differences are small in absolute terms when compared with trophic skew at high extents and intensities (figure 2).

### (b) Heterotroph response to fragmentation

**Figure 3.**
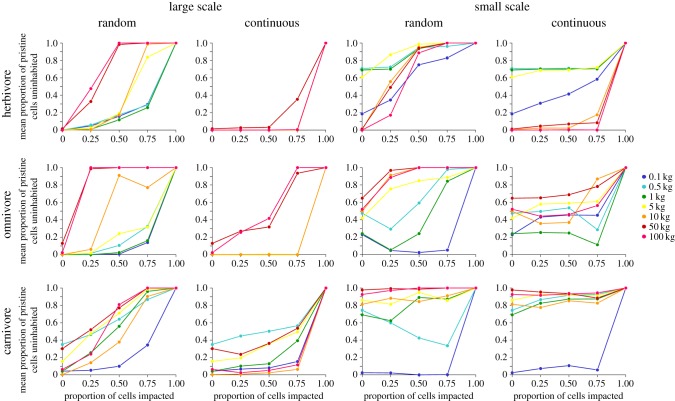
Representative subset of heterotroph responses (measured as the mean proportion of unimpacted cells that became uninhabited) to increasing extents of habitat loss under different spatial configurations and scales, separated by trophic group (panels) and body mass (line colour). Some lines exactly overlay each other, where populations only declined at total ecosystem collapse. Body masses across simulations ranged from <1 mg to 900 kg, with the above subset showing representative responses.

We investigated the responses of heterotroph populations by examining simulations with complete loss of patches (100% impact intensity), allowing us to label habitat patches as ‘suitable’ or ‘unsuitable’ without assumptions about patch quality. Populations followed a variety of responses to increasing spatial extent of habitat loss (figure 3), but were broadly consistent in showing higher probabilities of absence as impact extent increased. However, the exact form of the decline depended on both scenario and ecological factors—notably simulation scale, spatial configuration, and heterotroph traits including trophic group and body mass.

We identified complex interactions governing heterotroph responses (electronic supplementary material, S1 and figure 3). We clarified these interactions using our subset analyses (electronic supplementary material, S1 and table S2). Generally, severe population declines of large animals were observed at lower extents of habitat loss compared with smaller animals. In large-scale simulations, there was a marked difference in the extent of impact at which rapid declines occurred depending on the spatial configuration of impact. Population declines occurred at lower extents (less habitat removed) under random configurations compared with continuous, pointing to a negative effect of random spatial configuration. This elevated sensitivity to random configurations was more pronounced in larger animals.

At the small scale, populations are on average present in a smaller proportion of patches compared with the large scale, regardless of scenario. However, as at the large scale, large animals exhibit a higher sensitivity to random configurations compared with continuous. Across both scales, patterns of response differed in shape across the trophic groups, with differences in trophic sensitivity depending upon scale and configuration (figure 3). For all trophic groups, however, fragmented habitats showed comparatively more rapid population declines, and larger heterotrophs were more sensitive to impacts (electronic supplementary material, S1 and table S2).

### (c) Autotroph response to fragmentation

**Figure 4.**
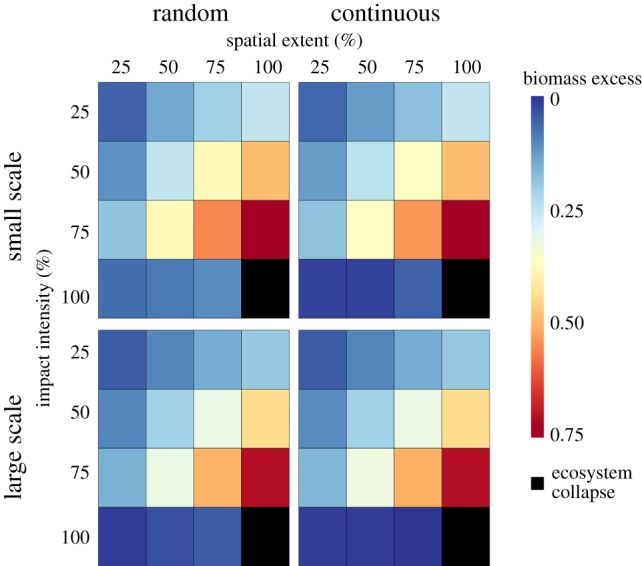
Mean gain in autotroph biomass as a result of top-down effects, as a proportion of the expected biomass assuming no top-down effects (‘biomass excess’) across different scenarios. Panels are split by simulation scale and spatial configuration. Black cells represent all plant biomass removed from the system.

Across our treatments, there was more autotroph biomass in the system than we would predict given the amount of plant biomass removed by our impacts (figure 4), indicating mitigation by top-down pressures of autotroph biomass loss. Mean levels of mitigation across the scenarios equalled 0.427 (42.7% more autotroph biomass than predicted).

Mitigation was slightly higher in the continuous configurations at the large scale (paired *t*-test, *t*_119_ = −4.91, *p* < 0.001, m.o.d. = −0.0451), corresponding to 4.51% more mitigation; however, at the small scale, the reverse was true: mitigation levels were slightly higher under random configurations (paired *t*-test, *t*_119_ = 5.68, *p* < 0.001, m.o.d. = 0.0513), corresponding to 5.13% more mitigation. Overall, small-scale simulations showed greater mitigation than at the large scale within all three configurations (paired *t*-tests; *continuous*: *t*_119_ = 5.80, *p* < 0.001, m.o.d. = 0.0403; *random: t*_119_ = 8.61, *p* < 0.001, m.o.d. = 0.137; *100% spatial extent: t*_69_ = 12.179, *p* < 0.001, m.o.d. = 0.0345), corresponding to a mean value of 7.50% more mitigation in the small-scale scenarios. As can be seen in figure 4, the size of these differences was very small compared with the size of the effect observed, with continuous and random configurations appearing almost identical.

Again, there were complex interactions governing mitigation (electronic supplementary material, S1). Directions of response were the same as trophic skew: there was stronger mitigation with increasing extent and intensity, with evidence of a synergistic effect. However, one main difference was apparent, there was little mitigation of plant biomass loss when intensity was 100% (figure 4 and electronic supplementary material, S1 and table S3).

## 4. Discussion

### (a) Ecosystem responses

We demonstrate profound effects on simulated ecosystems of land-use extent, intensity, fragmentation, and their interactions. There were complex interactions between all aspects of land use, leading to context-dependent differences in how the ecosystem responded. Further, different measures of ecosystem change responded differently to each aspect. For example, fragmentation exerted a strong influence on some heterotrophs but had a negligible effect on autotrophs.

#### (i) Trophic skew

Across our scenarios, trophic skew increased with both intensity and extent, indicating important differences in the responses of different trophic levels to land-use change. Our mean trophic skew value of 0.148 represents that following impact, a minimum of approximately 15% of the total ecosystem biomass was redistributed between autotrophs, herbivores, omnivores, and carnivores—a major change in ecosystem dynamics. This is not driven solely by autotroph biomass loss mitigation, as a high trophic skew is observed at 100% impact intensity, where the mitigation effect is not apparent (figures 2 and 4). Differences in heterotroph feeding guild responses to habitat change are well documented. Predators are known to be more sensitive to habitat loss [26,27,30]. In our simulations, greater sensitivity to habitat change among predators may compound with body size also affecting sensitivity. Predators are typically between 0.5 and 4 orders of magnitude larger than their prey [40], and this size structuring strongly influences trophic network behaviour [41,42]. Loss of predators, and larger animals more generally, may therefore strongly alter biomass flows.

Across simulated scenarios, increasing extent and intensity had more adverse effects on ecosystems where habitat was fragmented, compared with where habitat was contiguous. Our trophic skew metric, which we introduced as a coarse measure of ecosystem change, indicated that fragmentation exacerbated the effects of habitat loss by a mean value of 18.1% across scenarios. While this cannot be strictly compared with metrics of direct conservation concern (e.g. additional species extinctions), it can inform the likely magnitude of such figures. The mechanisms underlying this exacerbation should be considered.

Fragmentation more strongly affects higher trophic levels [23,28,43]. The potential synergy observed between loss and fragmentation could be driven by responses of heterotrophs in the system. Larger animals were identified as more sensitive to increasing impact severity, and that this sensitivity was heightened under random spatial configurations. A combination of mechanisms within the model could be mediating this increased sensitivity, including lower food availability leading to reduced fecundity, and increased need for dispersal and therefore greater ‘home range’ sizes. The Madingley model's dynamic and emergent nature means these processes do not act in isolation, and we cannot decisively say which simulated process leads proximately to population declines. Preferential loss of large animals is known to change ecosystem function [44,45]. Additionally, sequential extinctions ordered by body size have disproportionately major effects on ecosystem functioning [46]. If extinctions are happening in size sequence, then trophic skew will increase nonlinearly with the number of extinctions, a pattern that we observed in our model outputs. Fragmentation may therefore only need to marginally increase the number of size-ordered extinctions caused by habitat loss for there to be a much larger trophic skew, explaining the importance of fragmentation in this instance.

We also identified that smaller habitat landscapes appear to be more sensitive to impacts, particularly in the context of low or moderate levels of impact. Small-scale simulations yielded higher trophic skews than at the large scale, pointing to increased ecosystem resilience when considering larger habitat landscapes, especially in response to low levels of land-use change. This may be analogous to the identified role of larger home ranges increasing extinction risk [47], where even at low levels of impact the smaller-scale ecosystems dropped below a minimum absolute habitat size/quality to sustain the system's largest fauna—the ‘extinction threshold’ [7]. While home ranges are not an explicit in the model, analogous spatial requirements emerge as a result of the underlying simulated ecology. The apparent whole-ecosystem difference in sensitivity between landscapes sizes may contribute to debates on protected area design, favouring ‘single large’ over ‘several small’ reserves [48]. Further investigation into this apparent effect may benefit from comparing habitat landscapes where individual cell areas are the same for both large and small landscapes, unlike in our study where cell number remains constant. It is not necessarily the case that the model will behave equivalently when simulating large landscapes with much smaller cell sizes, should such an approach be computationally feasible.

#### (ii) Heterotroph response

Trophic groups differed in their responses to habitat loss and fragmentation, consistent with our trophic skew analysis (figure 2); potential mechanisms for this have been discussed above. Populations of animals of larger body size declined at relatively lower impact levels (figure 3 and electronic supplementary material, S1 and table S2). Larger animals are known to be more extinction prone [47,49]. This may be because larger animals reached their extinction threshold earlier as they require a larger absolute expanse of habitat to sustain stable populations [7].

Heterotroph populations generally declined more in the face of fragmented habitat loss (figure 3; electronic supplementary material, S1 and table S2). Again, this response was dependent on other traits and scenario-specific aspects of land use. This may be because beyond a certain level of fragmentation, habitat patches become too difficult to reach via dispersal and are functionally lost [7]. This explanation could account for the observation that the negative effect of higher body mass was more severe under fragmented configurations, as has been shown before [22,31,50]. Notably, the disproportionate sensitivity to fragmentation of large organisms was more apparent at the large scale (figure 3). Despite the larger scale maintaining greater absolute amounts of habitat, distances between isolated patches are greater, and dispersal between them therefore less likely. The fragmentation threshold will therefore be reached at comparably less severe impact scenarios, a problem for the largest animals in the system that are less likely to maintain stable populations within one single patch. In the specific context of large fauna, this mechanism may mean fragmentation is most relevant at larger landscape scales.

#### (iii) Autotroph response

There was more plant biomass present in ecosystems than expected, based on the amount removed under land-use change, across the majority of our scenarios (figure 4). Plant biomass was up to 1.7× the amount predicted by our ‘naive’ approach. This mitigation of autotroph biomass loss can only be explained by top-down trophic interactions because of the structure of the model simulations. Overall, the pattern of strength of mitigation was similar to our measure of trophic skew (figures 2 and 4), and again exhibited complex interactions (electronic supplementary material, S1). However, there were some differences. While there were significant differences in autotroph response when comparing between spatial configurations and scales modelled (electronic supplementary material, S1 and table S3), the magnitude of these differences was small (figure 4). Further, unlike our trophic skew results, mitigation was absent at 100% intensity (figure 4), likely because there were no autotrophs or heterotrophs remaining in these impacted patches. This observation shows that the simulated ecology driving this mitigation is largely happening within the impacted cells, rather than the remaining pristine parts of the ecosystem. This further explains why spatial configuration has little influence on the mitigation effect, as the mechanism responsible operated within impacted patches. Understood ecologically, this suggests that fragmentation is broadly unimportant compared with degree of habitat degradation in governing predicted mitigation.

### (b) Effects of model complexity

The Madingley model is a complex simulation of ecosystems, yet the metrics used in this study are coarse compared with the measures of ecosystem change possible in empirical studies. Given that the Madingley model demonstrates synergistic impacts of land use, using these coarse measures, our results suggest these synergies are more likely to occur empirically. Our findings can therefore provide insights on the way we study the impacts of fragmentation. Prior work has questioned whether some studies appropriately differentiate between habitat fragmentation and habitat loss [13,14]. Our comparisons of scenarios where habitat loss is equal, but spatial configuration different, separated these effects to show that fragmentation is broadly detrimental, and at a magnitude of clear ecological relevance. Prior theoretical work has predicted exacerbated extinction when habitat loss leaves many small habitat patches, compared with area-only calculations [8–10], and our findings are in agreement. Further, our findings point to strong synergy between fragmentation and other aspects of land-use change. This interdependence of effects supports addressing habitat fragmentation holistically [14].

Having demonstrated the potential importance of fragmentation in mediating responses to land-use change, future simulation work could benefit from comparison of more realistic patterns of fragmentation at different landscape scales. Much higher grid resolutions than used in this study are required to appropriately capture more realistic fragmentation patterns. Such studies will become computationally more feasible with time. Landscape-scale patterns of land-use change that would lend themselves to investigation using this approach are apparent in the scientific literature; for example, the potential impacts of Africa's ‘development corridors’ [51].

Additionally, the consideration of trophic interactions within the Madingley model allows us to better differentiate how different organisms respond to fragmentation, and how the synergy described above may, in part, be mediated by indirect trophic effects following land-use change. Our approach enables us to examine population vulnerability as part of a trophic network, potentially incorporating sequential extinctions which may be a mechanism underpinning observed synergies between aspects of habitat loss.

Consideration of heterotroph organisms in the Madingley model also allowed us to predict the mitigation of plant biomass loss following land-use change, caused by reduced top-down pressure. Notably, the size of this effect was large, supporting calls to integrate more ecology into earth-system models [52,53]. A consideration of trophic dynamics is likely to become increasingly important as land-surface models are required to operate on smaller scales [54], and our results highlight the potential magnitude of the feedback that heterotroph organisms may have on plant dynamics at these smaller scales.

Overall, our novel use of a general ecosystem model to study the effects of different aspects of land-use change on ecosystem structure contributes to current debates on how we best address habitat fragmentation. We demonstrate the likely negative effects of habitat fragmentation, which disproportionately affects animals of larger body size, and is particularly disruptive in less extensive ecosystems. Further, we identify clear interdependence of the effects of fragmentation and habitat loss, showing that the effects of these two aspects of land-use change should be assessed together. We further show that top-down effects of animals on plant biomass are likely to be important in determining vegetation structure in disturbed habitats, warranting consideration in both ecological and carbon stock modelling.

## Supplementary Material

Supplementary Material S1

## Supplementary Material

Supplementary Material S2

## Data accessibility

Input files required to recreate simulations in the open-access Madingley model are provided (electronic supplementary material, S2).
