# Supplementary material for: Synergistic impacts of habitat loss and fragmentation on model ecosystems
Source: Proc Biol Sci. 2016 Sep 28;283(1839):20161027. doi: 10.1098/rspb.2016.1027 (PMC5046893; doi:10.1098/rspb.2016.1027)
Supplement: Supplementary Material S1 [file rspb20161027supp1.pdf]

Supplementary Material 1

Madingley Model Representation

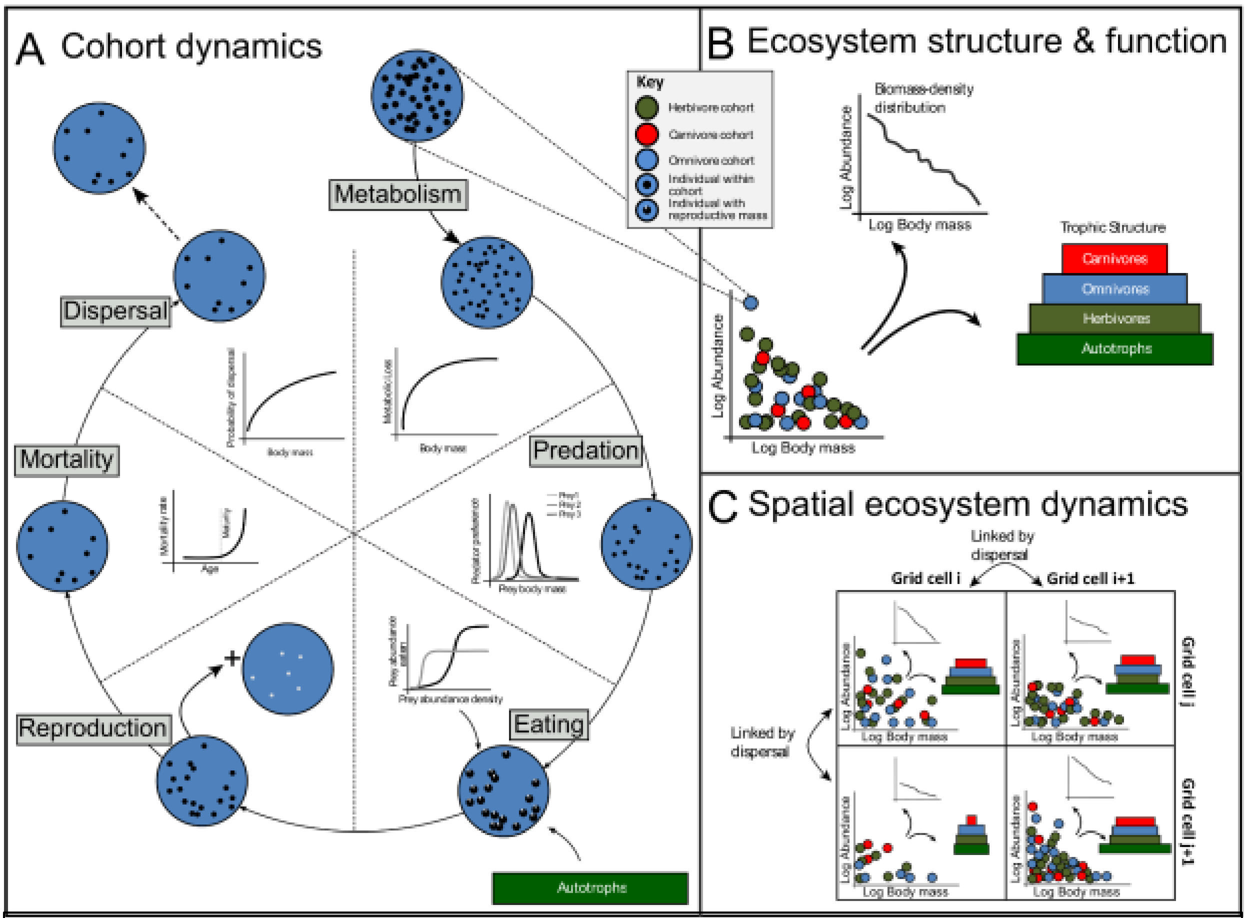

Figure S1: Diagrammatic representation of ecosystem structure and function. Ecosystem emerges from the combination of processes operating on individual organisms within a grid cell and dispersal between grid cells. Coloured circles represent cohorts of near-identical individuals, grouped together for computational efficiency. **Image from Harfoot, M.B.J., Newbold, T., Tittensor, D.P., Emmott, S., Hutton, J., Lyutsarev, V., *et al.* (2014). Emergent Global Patterns of Ecosystem Structure and Function from a Mechanistic General Ecosystem Model. *PLoS Biol.*, 12, e1001841.**

Summary of Land Use Scenarios Modelled

Table S1: Summary of the combinations of treatments simulated, totalling 29 scenarios. No distinction in spatial configuration of impact when extent is 0% or 100% as all cells are impacted equally. Each scenario listed was modelled with 10 replicates at both our large and small scales.

Proportion of Habitat Impacted (Extent)	Proportion of Autotroph Biomass Removed from Impacted Cells (Intensity)	Spatial Configuration
0.00	0.00	Not Applicable
0.25	0.25	Random, Continuous
0.25	0.50	Random, Continuous
0.25	0.75	Random, Continuous
0.25	1.00	Random, Continuous
0.50	0.25	Random, Continuous
0.50	0.50	Random, Continuous
0.50	0.75	Random, Continuous
0.50	1.00	Random, Continuous
0.75	0.25	Random, Continuous
0.75	0.50	Random, Continuous
0.75	0.75	Random, Continuous
0.75	1.00	Random, Continuous
1.00	0.25	Not Applicable
1.00	0.50	Not Applicable
1.00	0.75	Not Applicable
1.00	1.00	Not Applicable

Example Land Use Scenarios

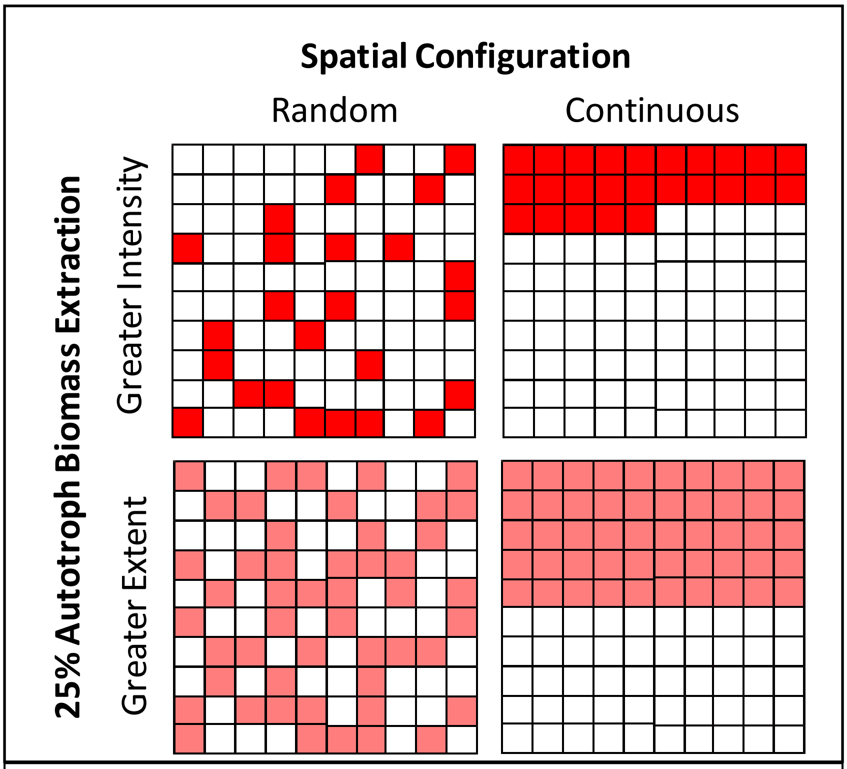

Figure S2: Examples of different treatments all extracting 25% of the total autotroph biomass from the system. Top row shows land-use intensity set to 1.0 and extent set to 0.25; bottom row shows intensity at 0.5 and extent at 0.5. Columns show random or continuous spatial configuration.

Linear Models: Trophic Skew

Initial Model – Generalised Linear Model, Normal Error Structure.

Model1 <- glm(TCM ~ ImpInt * SpatExt * SpatCon * Scale, family=gaussian)

Key: TCM – Trophic Skew; ImpInt – Impact Intensity; SpatExt – Spatial Extent; SpatCon – Spatial Configuration; Scale – Modelled Scale

Refinement Using ANOVA Model Comparisons

Minimum adequate model using this approach:

TCM ~ ImpInt + SpatExt + SpatCon + Scale + ImpInt:SpatExt + ImpInt:SpatCon + ImpInt:Scale + SpatExt:SpatCon + ImpInt:SpatExt:SpatCon

Refinement Using AIC Measures

Use of package 'MASS' (R, v 3.1.1), StepAIC(Model1) results in Minimum Adequate Model:

TCM ~ ImpInt + SpatExt + SpatCon + Scale + ImpInt:SpatExt + ImpInt:SpatCon + SpatExt:SpatCon + ImpInt:Scale + SpatExt:Scale + SpatCon:Scale + ImpInt:SpatExt:SpatCon + ImpInt:SpatCon:Scale + SpatExt:SpatCon:Scale

Linear Models: Heterotroph Response

Initial Models – Generalised Linear Model, quasibinomial Error Structure. Large Scale and Small Scale separated after viewing graphical differences.

LS/SSModel1 <- glm(formula = cbind(Inhab, Empty) ~ PropImp * SpatCon * MassBin * TrophicGroup, family=quasibinomial, data = LS/SS)

Key: Inhab – Counts of pristine cells inhabited; Empty – Counts of pristine cells uninhabited; PropImp – Spatial Extent; SpatCon – Spatial Configuration; TrophicGroup – Trophic Group; LS – Large Scale; SS – Small Scale

Refinement Using ANOVA Model Comparisons

Large Scale:

Test removal of most complex interaction (Large Scale):

LSModel1.1 <- update(LSModel1, ~ . - PropImp:SpatCon:MassBin:TrophicGroup)

ANOVA comparison between LSModel1 and LSModel1.1, **significant difference** (p < 0.001).

Most complex term cannot be dropped, most complicated model also minimum adequate.

Small Scale:

Summary of minimum adequate model:

cbind(Inhab, Empty) ~ PropImp + SpatArr + MassBin + TrophicGroup + PropImp:SpatArr + PropImp:MassBin + SpatArr:MassBin + PropImp:TrophicGroup + SpatArr:TrophicGroup + MassBin:TrophicGroup + PropImp:SpatArr:MassBin + PropImp:SpatArr:TrophicGroup + PropImp:MassBin:TrophicGroup + SpatArr:MassBin:TrophicGroup

Subsetted Models: Generlised-Linear Mixed-Effects Models

Of the form: Model <- glmer(formula = cbind(Inhab, Empty) ~ PropImp + MassBin + (1|TrophicGroup), family=binomial, data = <appropriate subset>)

Table S2: Regression coefficients and significance for fixed effects in GLMM subsets. Response variable was proportion of pristine cells remaining inhabited. Subsets split by simulation scale and spatial configuration, with impact intensity set to 100%.

Subset	Fixed Effect	Coefficient	p-value
**Large Scale, Random**			
	Spatial Extent	-5.159	< 0.001
	Mass	-0.466	< 0.001
**Large Scale, Continuous**			
	Spatial Extent	-1.608	< 0.001
	Mass	-0.193	< 0.001
**Small Scale, Random**			
	Spatial Extent	-2.340	< 0.001
	Mass	-0.255	< 0.001
**Small Scale, Continuous**			
	Spatial Extent	-0.558	< 0.001
	Mass	-0.020	< 0.001

Linear Models: Autotroph Response

Initial Model – Generalised Linear Model, Normal Error Structure.

Model1 <- glm(DeltaBiomass ~ ImpInt * SpatExt * SpatCon * Scale, family=gaussian)

Key: DeltaBiomass – Difference in Expected and Observed Autotroph Biomass; ImpInt – Impact Intensity; SpatExt – Spatial Extent; SpatCon – Spatial Configuration; Scale – Modelled Scale

Refinement Using ANOVA Model Comparisons

Minimum adequate model using this approach:

DeltaBiomass ~ ImpInt + SpatExt + SpatArr + Scale + ImpInt:SpatArr + ImpInt:Scale + SpatArr:Scale + ImpInt:SpatArr:Scale

Refinement Using AIC Measures

Use of package 'MASS' (R, v 3.1.1), StepAIC(Model1) results in Minimum Adequate Model:

DeltaBiomass ~ ImpInt + SpatExt + SpatCon + Scale + ImpInt:SpatExt + ImpInt:SpatCon + ImpInt:Scale + SpatExt:Scale + SpatCon:Scale + ImpInt:SpatExt:Scale + ImpInt:SpatCon:Scale

Table S3. Regression analyses performed on data subsets looking at autotroph biomass responses. Subset value represents the value of either extent or intensity for that subset, depending on which variable the regressions was performed on. β is the regression coefficient for autotroph biomass difference against either extent or intensity (see column headings). Intensity regressions excluded 100% intensity.

	Large Scale								Small Scale							
	Extent Regression (subset by intensity)				Intensity Regression (subset by extent)				Extent Regression (subset by intensity)				Intensity Regression (subset by extent)			
**Subset Value**	β	R^2	d.f.	p	B	R^2	d.f.	p	β	R^2	d.f.	p	β	R^2	d.f.	p
**0.25**	0.351	0.969	78	< 0.001	0.385	0.967	58	< 0.001	0.383	0.910	78	< 0.001	0.416	0.906	58	< 0.001
**0.50**	0.539	0.996	78	< 0.001	0.580	0.994	58	< 0.001	0.562	0.985	78	< 0.001	0.566	0.964	58	< 0.001
**0.75**	0.786	0.998	78	< 0.001	0.802	0.998	58	< 0.001	0.771	0.997	78	< 0.001	0.755	0.987	58	< 0.001
**1.00**	-0.289	0.170	58	< 0.001	1.10	0.999	58	< 0.001	0.204	0.087	58	< 0.001	1.00	0.998	58	< 0.001
