# Supplementary material for: Synergistic impacts of habitat loss and fragmentation on model ecosystems
Source: Proc Biol Sci. 2016 Sep 28;283(1839):20161027. doi: 10.1098/rspb.2016.1027 (PMC5046893; doi:10.1098/rspb.2016.1027)
Supplement: Supplementary Material S2 [file rspb20161027supp2.pdf]

Supplementary Material 2

Madingley Model Input Files

The following tables detail the input files and parameters used which would could be used in conjunction with the Madingley Model to exactly recreate our experimental runs. Whilst we explain the basics of the information provided here, they should be interpreted in the context of Harfoot et al. (2014) [1], particularly in the case of parameter values (Table S6).

Table S3. **The scenarios file ('Scenarios.csv') which defined our treatments in both the small scale and large scale simulations. 'label' specifies the file outputs names. 'Spatial configuration' includes both random /continuous configuration and the spatial extent. 'npp' equated to intensity (proportion plant biomass harvested). Simulation number defines replicates. 'temperature' and 'harvesting' were not used in this study.**

label	spatial configuration	npp	temperature	harvesting	simulation number
NI	no 0	no 0.0	no 0.0	no 0.0	10
NPPCO2T10	continuous 0.25	temporary 1.0	no 0.0	no 0.0	10
NPPCO2T2	continuous 0.25	temporary 0.25	no 0.0	no 0.0	10
NPPCO2T5	continuous 0.25	temporary 0.5	no 0.0	no 0.0	10
NPPCO2T7	continuous 0.25	temporary 0.75	no 0.0	no 0.0	10
NPPCO5T10	continuous 0.5	temporary 1.0	no 0.0	no 0.0	10
NPPCO5T2	continuous 0.5	temporary 0.25	no 0.0	no 0.0	10
NPPCO5T5	continuous 0.5	temporary 0.5	no 0.0	no 0.0	10
NPPCO5T7	continuous 0.5	temporary 0.75	no 0.0	no 0.0	10
NPPCO7T10	continuous 0.75	temporary 1.0	no 0.0	no 0.0	10
NPPCO7T2	continuous 0.75	temporary 0.25	no 0.0	no 0.0	10
NPPCO7T5	continuous 0.75	temporary 0.5	no 0.0	no 0.0	10
NPPCO7T7	continuous 0.75	temporary 0.75	no 0.0	no 0.0	10
NPPCO10T2	continuous 1.0	temporary 0.25	no 0.0	no 0.0	10
NPPCO10T5	continuous 1.0	temporary 0.5	no 0.0	no 0.0	10
NPPCO10T7	continuous 1.0	temporary 0.75	no 0.0	no 0.0	10
NPPRN2T10	random 0.25	temporary 1.0	no 0.0	no 0.0	10
NPPRN2T2	random 0.25	temporary 0.25	no 0.0	no 0.0	10
NPPRN2T5	random 0.25	temporary 0.5	no 0.0	no 0.0	10
NPPRN2T7	random 0.25	temporary 0.75	no 0.0	no 0.0	10
NPPRN5T10	random 0.5	temporary 1.0	no 0.0	no 0.0	10
NPPRN5T2	random 0.5	temporary 0.25	no 0.0	no 0.0	10
NPPRN5T5	random 0.5	temporary 0.5	no 0.0	no 0.0	10
NPPRN5T7	random 0.5	temporary 0.75	no 0.0	no 0.0	10
NPPRN7T10	random 0.75	temporary 1.0	no 0.0	no 0.0	10
NPPRN7T2	random 0.75	temporary 0.25	no 0.0	no 0.0	10
NPPRN7T5	random 0.75	temporary 0.5	no 0.0	no 0.0	10
NPPRN7T7	random 0.75	temporary 0.75	no 0.0	no 0.0	10
NPPRN10T2	random 1.0	temporary 0.25	no 0.0	no 0.0	10
NPPRN10T5	random 1.0	temporary 0.5	no 0.0	no 0.0	10
NPPRN10T7	random 1.0	temporary 0.75	no 0.0	no 0.0	10
NPPRN10T10	random 1.0	temporary 1.0	no 0.0	no 0.0	10

Table S4. Model initialisation file ('EcosystemModelinitialisation.csv'). Relevant short descriptions of the inputs are given. The rows which are relevant to our results and changed to adjust between small scale and large scale simulations are highlighted.

Parameter	Value (Large Scale)	Value (Small Scale)	Description	Relevant to results?
Timestep Units	Month	Month	Size of timestep to be used in the simulation.	
Length of simulation (years)	100	100	Length of simulation from end of burn-in (years).	Yes
Burn-in (years)	100	100	Length of time given for simulations to equilibrate before 'main' simulation begins (years).	Yes
Impact duration (years)	100	100	Length of time impacts should be enacted for (< or = to length of simulation, years).	Yes
Bottom Latitude	0	0.5	Southernmost latitude of grid (decimal degrees).	Yes
Top Latitude	1	0.6	Northernmost latitude of grid (decimal degrees).	Yes
Leftmost Longitude	37.5	38	Westernmost longitude of grid (decimal degrees).	Yes
Rightmost Longitude	38.5	38.1	Easternmost longitude of grid (decimal degrees).	Yes
Grid Cell Size	0.1	0.01	Length of one side of grid cell (decimal degrees).	Yes
Grid Cell Rarefaction	1	1	Not applicable to this study (1 is no rarefaction).	
Run Cells In Parallel	no	no	Assigns different cells to different processor cores.	
Run Simulations In Parallel	yes	yes	Assigns different simulation runs to different processor cores.	
Run Single Realm			Not applicable to this study, area only included terrestrial realm.	
Extinction Threshold	1	1	Number of individuals remaining in a cohort for it be considered extant (>= 1).	
Maximum Number Of Cohorts	1000	1000	The maximum number of cohorts allowed within a single cell, after which the model merges the most similar cohorts into single larger approximations.	
Output Filename	FinalOutputs	FinalOutputs	Assigns a filname to which output files are written.	
Track Processes	no	No	Tracks details of ecological processes occurring in the model. Computationally expensive.	
Track Global Processes	no	No	Outputs global summaries of the processes which occurred in the model each time step, including Net Primary Productivity (NPP).	
Read State			Not applicable for this study (used to start simulation from a previous simulation output).	
Mass Bin Filename	MassBinDefinitions.csv	MassBinDefinitions.csv	Name of file with mass bin definitions for model outputs.	
New Cohorts Filename	NewCohorts	NewCohorts	Name of file governing reproduction processes.	
Maturity Filename	Maturity	Maturity	Name of file governing maturation processes.	
Predation Flows Filename	PredationFlows	PredationFlows	Name of file governing predation processes.	
Herbivory Flows Filename	HerbivoryFlows	HerbivoryFlows	Name of file governing herbivory processes.	
Biomasses Eaten Filename	BiomassesEaten	BiomassesEaten	Name of file governing biomass tracking processes.	
Trophic Flows Filename	TrophicFlows	TrophicFlows	Name of file governing trophic flow tracking processes.	
Mortality Filename	Mortality	Mortality	Name of file governing mortality processes.	
Extinction Filename	Extinction	Extinction	Name of file governing extinction tracking processes.	
Growth Filename	Growth	Growth	Name of file governing heterotroph growth processes.	
Metabolism Filename	Metabolism	Metabolism	Name of file governing heterotroph metabolism processes.	
NPP Filename	NPP	NPP	Name of file governing autotroph growth processes.	
Environmental Data File	EnvironmentalDataLayers.csv	EnvironmentalDataLayers.csv	Name of file directing to climate / environmental data layers.	
Specific Location File			Not applicable to this study (used in stead of longitude / latitude above).	
Cohort Functional Group Definitions File	CohortFunctionalGroupDefinitions.csv	CohortFunctionalGroupDefinitions.csv	Name of file containing functional group definitions for the simulated heterotrophs.	
Stock Functional Group Definitions File	StockFunctionalGroupDefinitions.csv	StockFunctionalGroupDefinitions.csv	Name of file containing functional group definitions for the simulated autotrophs.	
Output Detail	high	high	Amount of information to be recorded by the model. 'high' shows cell-by-cell-by-timestep split. Biomass and abundance resolved to functional groups and mass bins.	
Dispersal only	no	no	Run only dispersal aspect of the model. Not used for experimental work.	
Dispersal only type			Not relevant to this study (see 'no' above).	
Plankton size threshold	0.01	0.01	Not relevant to this study (no marine realm).	
Live outputs	no	no	Runs live visualisation of models.	
Track marine specifics	no	no	Not relevant to this study (no marine realm).	
Output metrics	yes	yes	Should model outputs be written (combines with output detail above).	
Output model state timesteps	no	no	Should separate model outputs be written for each time stamp.	

Table S5. Parameter table showing values from the version of Madingley used in this study. It should be noted that this is not an initialization file, but simple a log of the fundamental parameters coded into Madingley and was not edited for the purpose of this study. We show it here for reference against future studies in which these values may be different. Explanations of these parameters are found in Harfoot et al 2014 [1].

Ecological process	Parameter name	Parameter value
Herbivory	TimeUnitImplementation	Day
Herbivory	ReferenceMass_g	1
Herbivory	HandlingTimeScalarTerrestrial	0.7
Herbivory	HandlingTimeScalarMarine	0.7
Herbivory	HandlingTimeExponentTerrestrial	0.7
Herbivory	HandlingTimeExponentMarine	0.7
Herbivory	HerbivoryRateConstant	1.00E-11
Herbivory	_AttackRateExponentTerrestrial	1
Herbivory	_AttackRateExponentMarine	1
Herbivory	HerbivoryRateMassExponent	1
Predation	TimeUnitImplementation	Day
Predation	ReferenceMass_g	1
Predation	HandlingTimeScalarTerrestrial	0.5
Predation	HandlingTimeExponentTerrestrial	0.7
Predation	HandlingTimeScalarMarine	0.5
Predation	HandlingTimeExponentMarine	0.7
Predation	KillRateConstant	1.00E-06
Predation	FeedingPreferenceStandardDeviation	0.7
Predation	KillRateConstantMassExponent	1
Endothermic Metabolism	TimeUnitImplementation	day
Endothermic Metabolism	MetabolsimMassExponent	0.7
Endothermic Metabolism	NormalizationConstant	9.08091E+11
Endothermic Metabolism	ActivationEnergy_eV	0.69
Endothermic Metabolism	BoltzmannConstant_eV_per_K	8.62E-05
Endothermic Metabolism	EnergyScalar_kJ_to_g	0.036697248
Endothermic Metabolism	BodyTemperature_K	310
Ectothermic Metabolism	TimeUnitImplementation	day
Ectothermic Metabolism	MetabolismMassExponent	0.88
Ectothermic Metabolism	NormalizationConstant	1.48984E+11
Ectothermic Metabolism	ActivationEnergy_eV	0.69
Ectothermic Metabolism	BoltzmannConstant_eVperK	8.62E-05
Ectothermic Metabolism	EnergyScalar_kJ_to_g	0.036697248
Ectothermic Metabolism	NormalizationConstantBMR	41918272883
Ectothermic Metabolism	BasalMetabolismMassExponent	0.69
Background Mortality	TimeUnitImplementation	Day
Background Mortality	MortalityRate	0.001
Senescence Mortality	TimeUnitImplementation	Day
Senescence Mortality	MortalityRate	0.003
Starvation Mortality	TimeUnitImplementation	Day
Starvation Mortality	LogisticInflectionPoint	0.6
Starvation Mortality	MaximumStarvationRate	1
Starvation Mortality	LogisticScalingParameter	0.05
Reproduction	TimeUnitImplementation	month
Reproduction	MassRatioThreshold	1.5
Reproduction	MassEvolutionProbability	0.95
Reproduction	MassEvolutionStandardDeviation	0.05
Reproduction	SemelparityAdultMassAllocation	0.5
Diffusive Dispersal	TimeUnitImplementation	month
Diffusive Dispersal	DispersalSpeedBodyMassScalar_per_g	0.0278
Diffusive Dispersal	DispersalSpeedBodyMassExponent	0.48

Terrestrial Plant Model	max_NPP	0.961644704
Terrestrial Plant Model	t1_NPP	0.237468183
Terrestrial Plant Model	t2_NPP	0.100597089
Terrestrial Plant Model	p_NPP	0.001184101
Terrestrial Plant Model	FracStructScalar	7.154615419
Terrestrial Plant Model	a_FracEvergreen	1.7
Terrestrial Plant Model	b_FracEvergreen	-1.828591558
Terrestrial Plant Model	c_Frac_Evergreen	0.8
Terrestrial Plant Model	m_EGLeafMortality	0.040273936
Terrestrial Plant Model	c_EGLeafMortality	1.013070062
Terrestrial Plant Model	m_DLeafMortality	0.020575964
Terrestrial Plant Model	c_DLeafMortality	-1.195235464
Terrestrial Plant Model	m_FRootMort	0.04309283
Terrestrial Plant Model	c_FRootMort	-1.478393163
Terrestrial Plant Model	p2_StMort	0.139462774
Terrestrial Plant Model	p1_StMort	-4.395910091
Terrestrial Plant Model	MaxFracStruct	0.362742634
Terrestrial Plant Model	LFSHalfSaturation_Fire	0.388125108
Terrestrial Plant Model	LFSScalar_Fire	19.98393943
Terrestrial Plant Model	NPPHalfSaturation_Fire	1.148698636
Terrestrial Plant Model	NPPScalar_Fire	8.419032427
Terrestrial Plant Model	er_min	0.01
Terrestrial Plant Model	er_max	24
Terrestrial Plant Model	dr_min	0.01
Terrestrial Plant Model	dr_max	24
Terrestrial Plant Model	frm_min	0.01
Terrestrial Plant Model	frm_max	12
Terrestrial Plant Model	stm_max	1
Terrestrial Plant Model	stm_min	0.001
Terrestrial Plant Model	BaseScalar_Fire	2
Terrestrial Plant Model	MinReturnInterval	2.26E-06
Terrestrial Plant Model	CarbonToLeafDryMatterScalar	0.476
Terrestrial Plant Model	LeafDryMatterToLeafWetMatterScalar	0.213
Activity	TerrestrialWarmingToleranceIntercept	6.61
Activity	TerrestrialWarmingToleranceSlope	1.6
Activity	TerrestrialTSMIntercept	1.51
Activity	TerrestrialTSMSlope	1.53
Activity	MarineUpperToleranceIntercept	43.2
Activity	MarineUpperToleranceSlope	-0.14
Activity	MarineRangeIntercept	31.2
Activity	MarineRangeSlope	-0.13

Bibliography

1. Harfoot, M. B. J., Newbold, T., Tittensor, D. P., Emmott, S., Hutton, J., Lyutsarev, V., Smith, M. J., Scharlemann, J. P. W. & Purves, D. W. 2014 Emergent Global Patterns of Ecosystem Structure and Function from a Mechanistic General Ecosystem Model. *PLoS Biol.* **12**, e1001841. (doi:10.1371/journal.pbio.1001841)
